# Comparison of methylation capture sequencing and Infinium MethylationEPIC array in peripheral blood mononuclear cells

**DOI:** 10.1186/s13072-020-00372-6

**Published:** 2020-11-23

**Authors:** Chang Shu, Xinyu Zhang, Bradley E. Aouizerat, Ke Xu

**Affiliations:** 1 Department of Psychiatry, Yale School of Medicine, New Haven, CT 06516 USA; 2 Connecticut Veteran Healthcare System, West Haven, CT 06515 USA; 3 Bluestone Center for Clinical Research, College of Dentistry, New York University, New York, 10010 USA; 4 Department of Oral and Maxillofacial Surgery, College of Dentistry, Yale School of Medicine, New York University, New York, 10010 USA

**Keywords:** Methylation capture sequencing, EPIC, DNA methylation, Peripheral blood mononuclear cells

## Abstract

**Background:**

Epigenome-wide association studies (EWAS) have been widely applied to identify methylation CpG sites associated with human disease. To date, the Infinium MethylationEPIC array (EPIC) is commonly used for high-throughput DNA methylation profiling. However, the EPIC array covers only 30% of the human methylome. Methylation Capture bisulfite sequencing (MC-seq) captures target regions of methylome and has advantages of extensive coverage in the methylome at an affordable price.

**Methods:**

Epigenome-wide DNA methylation in four peripheral blood mononuclear cell samples was profiled by using SureSelectXT Methyl-Seq for MC-seq and EPIC platforms separately. CpG site-based reproducibility of MC-seq was assessed with DNA sample inputs ranging in quantity of high (> 1000 ng), medium (300–1000 ng), and low (150 ng–300 ng). To compare the performance of MC-seq and the EPIC arrays, we conducted a Pearson correlation and methylation value difference at each CpG site that was detected by both MC-seq and EPIC. We compared the percentage and counts in each CpG island and gene annotation between MC-seq and the EPIC array.

**Results:**

After quality control, an average of 3,708,550 CpG sites per sample were detected by MC-seq with DNA quantity > 1000 ng. Reproducibility of DNA methylation in MC-seq-detected CpG sites was high among samples with high, medium, and low DNA inputs (*r* > 0.96). The EPIC array captured an average of 846,464 CpG sites per sample. Compared with the EPIC array, MC-seq detected more CpGs in coding regions and CpG islands. Among the 472,540 CpG sites captured by both platforms, methylation of a majority of CpG sites was highly correlated in the same sample (*r*: 0.98–0.99). However, methylation for a small proportion of CpGs (*N* = 235) differed significantly between the two platforms, with differences in beta values of greater than 0.5.

**Conclusions:**

Our results show that MC-seq is an efficient and reliable platform for methylome profiling with a broader coverage of the methylome than the array-based platform. Although methylation measurements in majority of CpGs are highly correlated, a number of CpG sites show large discrepancy between the two platforms, which warrants further investigation and needs cautious interpretation.

## Introduction

The rapid increase in the number of epigenome-wide association studies (EWAS) have successfully identified differentially methylated CpG sites that are associated with environmental exposures and diseases [1–6]. Such DNA methylation marks have been used as biomarkers for diagnosing, subtyping, and monitoring disease progression [7–11]. The most popular and affordable methods to profile epigenome-wide DNA methylation are array-based platforms, primarily the Illumina Human Methylation 450 K (450 K) and Infinium MethylationEPIC (EPIC) BeadChips (Illumina Inc, San Diego, CA). These arrays utilize Illumina’s beadchip technology that does not require polymerase chain reaction (PCR), but is subject to dye intensity biases between the two platforms [12]. These arrays have limited coverage of the methylome and can only detect up to 870,000 CpGs across the epigenome, leaving a large proportion of CpG sites unmeasured. Moreover, the EPIC array offers improved but still suboptimal coverage of regulatory elements [13]. Whole-genome bisulfite sequencing (WGBS) is able to capture more than 28 million CpGs, but the feasibility remains low for the population-based EWAS due to high cost and large genomic DNA input requirements to compensate for degradation during DNA bisulfite treatment. Alternatively, Methylation Capture Sequencing (MC-seq) is able to detect DNA methylation at single-nucleotide resolution utilizing a targeted next-generation sequencing approach [14]. It permits profiling of significantly more CpG sites than the EPIC array, requires less genomic DNA input than WGBS, and less expensive than WGBS, but can be susceptible to bias due to the presence of PCR duplicates. Feature-to-cost comparisons among different platforms can help understand the utilities of each platform and provide guidance for investigators in choosing a methylation profiling platform.

A few studies have compared the CpG coverage, reproducibility, and performance of array-based and MC-seq platforms [15–17]. Teh et al. compared MC-seq and the 450 K array in seven DNA samples extracted from saliva [15]. A recent study compared the EPIC array and TruSeq targeted bisulfite sequencing in four cord blood DNA samples [17]. However, no comparisons of MC-seq and array-based methylome profiling of peripheral blood mononuclear cells (PBMCs) has been reported. Here, we profiled the DNA methylome in PBMCs using the Agilent SureSelect Methyl-Seq platform and compared the results to the EPIC array in DNA samples extracted from PBMCs.

## Methods

### Methylation capture sequencing (MC-seq)

#### DNA samples description

DNA was extracted from de-identified PBMCs collected from four individuals. Genomic DNA quality was determined by estimating the A260/A280 and A260/A230 ratios by spectrophotometry and concentration by fluorometry. DNA integrity and fragment size were confirmed using a microfluidic chip run on an Agilent Bioanalyzer. To assess the reproducibility of MC-seq by DNA quantity, DNA samples from each participant were profiled in triplicate times with high (> 1000 ng), medium (300–1000 ng), and low (150–300 ng) DNA input. In total, 12 DNA samples were measured by MC-seq. Bisulfate conversion was conducted for each DNA sample as described below.

#### Methyl-seq target enrichment library prep

Indexed paired-end whole-genome sequencing libraries were prepared using the SureSelect XT Methyl-Seq kit (Agilent, part#G9651B). Genomic DNA was sheared to a fragment length of 150–200 bp using focused acoustic energy delivered by the Covaris E220 system (Covaris, part#500003). Fragmented sample size distribution was determined using the Caliper LabChip GX system (PerkinElmer, Part#122000). Fragmented DNA ends were repaired with T4 DNA Polymerase and Polynucleotide Kinase and “A” base was added using Klenow fragment in a single reaction followed by AMPure XP bead-based purification (Beckman Coulter, part#A63882). The methylated adapters were ligated using T4 DNA ligase followed by AMPure XP bead purification. Quality and quantity of adapter-ligated DNA were assessed using the Caliper LabChip GX system. Samples yielding > 350 ng were enriched for targeted methylation sites by using the custom SureSelect Methyl-Seq Capture Library. Hybridization was performed at 65 °C for 16 h using a C1000 Thermal Cycler (BIO-RAD, part# 1851197). Once the enrichment was completed, the samples were mixed with streptavidin-coated beads (Thermo Fisher Scientific, part#65602) and washed with a series of buffers to remove non-specific bound DNA fragments. DNA fragments were eluted from beads with 0.1 M NaOH. Unmethylated C residues of enriched DNA were modified by bisulfite conversion using the EZ DNA Methylation-Gold Kit (Zymo Research, part#D5005). The SureSelect enriched, bisulfite-converted libraries were PCR amplified using custom-made indexed primers (IDT, Coralville, Iowa). Dual-indexed libraries were quantified by quantitative polymerase chain reaction (qPCR) using the Library Quantification Kit (KAPA Biosystems, Part#KK4854) and inserts size distribution was assessed using the Caliper LabChip GX system. Samples with a yield of ≥ 2 ng/μl were proceeded to sequencing.

#### Flow cell preparation and sequencing

Sample concentrations were normalized to 10 nM and loaded onto an Illumina NovaSeq flow cell at a concentration that yields 40 million passing filter clusters per sample. Samples were sequenced using 100 bp paired-end sequencing on an Illumina HiSeq NovaSeq according to Illumina standard protocol. The 10 bp dual index was read during additional sequencing reads that automatically follows the completion of the first read. Data generated during sequencing runs were simultaneously transferred to the Yale Center for Genome Analysis high-performance computing cluster. A positive control (prepared bacteriophage Phi X library) provided by Illumina was spiked into every lane at a concentration of 0.3% to monitor sequencing quality in real time.

#### Preprocessing and quality control

Signal intensities were converted to individual base calls during a run using the system’s Real Time Analysis (RTA) software. Sample de-multiplexing and alignment to the human genome was performed using Illumina’s CASAVA 1.8.2 software suite. The sample error rate was required to be less than 1% and the distribution of reads per sample in a lane was required to be within reasonable tolerance.

Quality control (QC) on MC-seq was conducted following standard procedure as previously described [18]. Quality of sequence data was examined by using *FastQC* (ver. 0.11.8). Adapter sequences and fragments at 5′ and 3′ (phred score < 20) with poor quality were removed by *Trim_galore* (ver. 0.6.3_dev). We used Bismark pipelines (ver. v0.22.1_dev) to align the reads to the bisulfite human genome (hg19) with default parameters [19]. Quality-trimmed paired-end reads were transformed into a bisulfite converted forward strand version (C → T conversion) or into a bisulfite-treated reverse strand (G → A conversion of the forward strand). Duplicated reads were removed from the Bismark mapping output by *deduplicate_bismark* and CpG, CHG, and CHH (where *H* = A, T, or C) were extracted by *bismark_methylation_extractor*.

All CpG sites were grouped by sequencing coverage, also known as read depth. The groups with coverage of 1× to 100× were used to test the relationship between coverage and number of CpG sites. Only the CpG sites with coverage > 10× depth were used for final comparisons to ensure MC-seq data quality. Genes were annotated using Homer *annotatePeaks.pl*, including intergenic, 5′UTR, promoter, exon, intron, 3′UTR, transcription start site (TTS), and non-coding categories. CpG island, shore, shelf, and open sea annotation were defined by locally developed bash and R scripts based on genomic coordinates (hg19) of CpG islands from the UCSC genome browser. CpG shores was defined as up to 2 kb from CpG islands and CpG shelf was defined as up to 2 kb from a CpG shore.

#### Assessment of reproducibility

We assessed CpG- and participant-based reproducibility for MC-seq among 12 samples with DNA quantity of high, medium, and low input in two ways. First, CpG-based reproducibility was assessed by calculating Pearson correlations using the CpG sites in common of the samples from the same participant with different input DNA quantities. Scatterplots were rendered showing 10,000 randomly selected common CpG sites comparing samples with high and medium, high and low, and medium and low DNA inputs. Second, participant-based reproducibility was assessed by comparing methylation profiles among pairs of participants using the samples with high DNA inputs, by calculating Pearson correlations of common CpG sites.

### EPIC array data preprocessing

The Infinium MethylationEPIC array (Illumina, San Diego, CA, USA) was used to measure PBMC DNA methylation profiles from the same four participants. These four samples with DNA input of 1000 ng were preprocessed using standard procedures as previously described [20]. Briefly, the predicted sex based on methylome was consistent with self-reported sex for all samples. All samples had a call rate greater than 0.15. A total of 19,090 CpG sites on X chromosomes and 537 CpG sites on Y chromosomes were filtered. A total of 846,464 CpG sites passed quality control.

### Comparison of methylation at each CpG site between MC-seq and EPIC array

The overall distribution of gene annotation in relation to CpG island and genetic region between MC-seq and EPIC array data from the four participants was compared. Common CpG sites between MC-seq and EPIC array assays were defined according to genomic coordinates. Pearson correlation and the absolute beta-difference value (Δ*β*) were calculated among common CpG sites between MC-seq methylation percentage values and EPIC methylation beta values by using R (ver. 3.5.1). If median Δ*β* of the common CpG site between two platforms was > 0.1, it was defined as a discordant CpG pair; otherwise, the CpG site was defined as a concordant CpG pair. The density plot of Δ*β* and a Manhattan plot showing the distribution of Δ*β* across epigenome were illustrated. Scatterplots were rendered showing the correlation of *β* values from 10,000 randomly selected CpG sites measured by both MC-seq and EPIC array.

## Results

### MC-seq overview and reproducibility

**Fig. 1:**
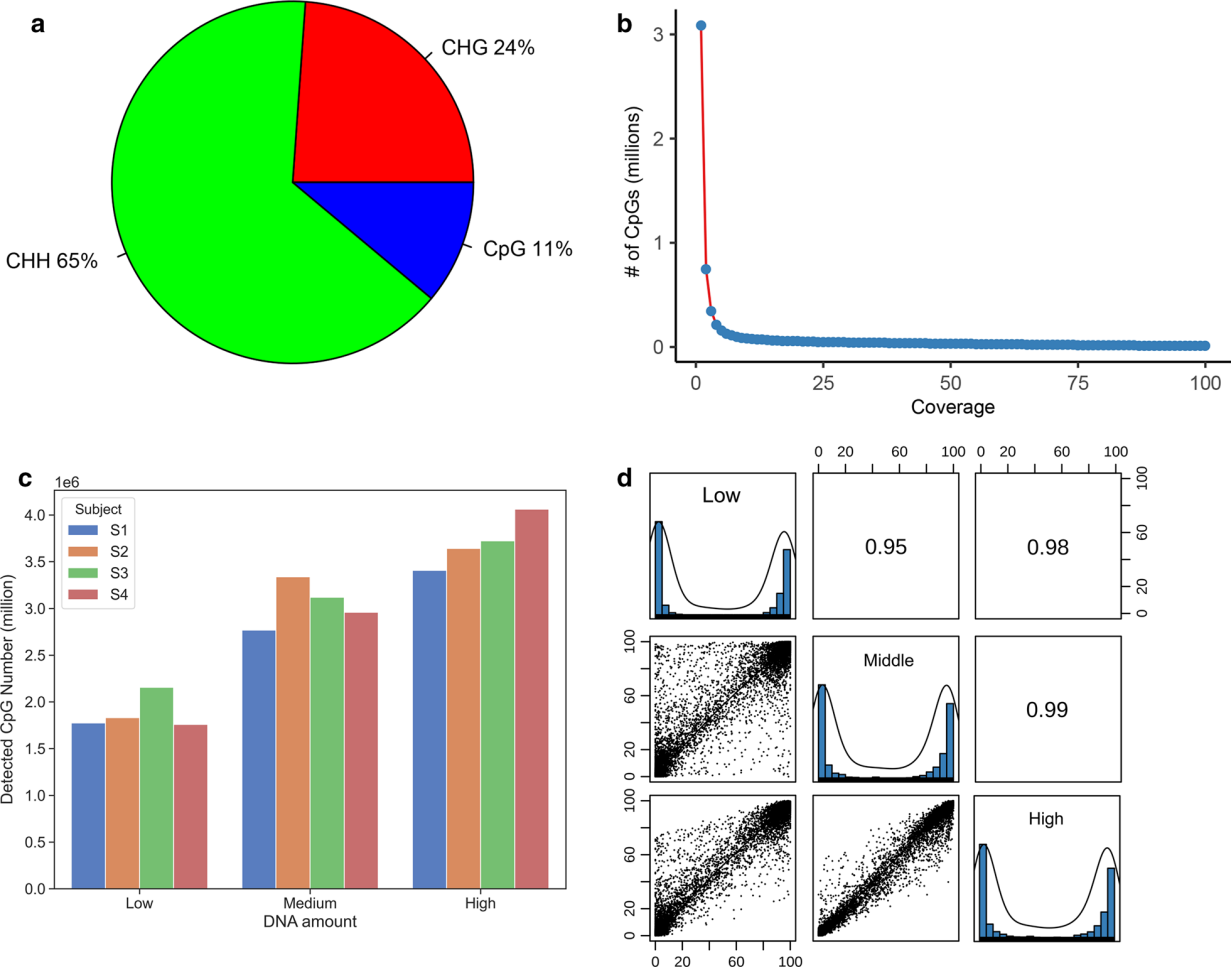
Methylation Capture Sequencing (MC-seq). **a** Distribution of methylation sequence context (CpG, CHH, CHG); **b** Coverage depth versus a number of detected CpG sites; **c** Detected CpG sites in low, medium, and high DNA inputs for four participants using MC-seq with minimum coverage ≥ 10×; **d** Scatterplots comparing 10,000 randomly selected common CpG sites among samples with high, medium, and low DNA input quantities and their Pearson correlations

In MC-seq, all sequences were efficiently mapped to the reference genome with greater than 89% mapping efficiency. Interestingly, the number of non-CpG sites was significantly greater than the number of CpG sites. Among all detected methylation sites by MC-seq, 11% were CpG sites, 65% were CHH sites, and 24% were CHG sites (Fig. 1a).

Figure 1b shows the relationship of the number of detected CpG sites and depth of sequence coverage by MC-seq in one sample. The depth of read at which the majority of sites were sequenced was estimated to be approximately 10× coverage, observed as the inflection point of the distribution of Fig. 1b. An increase of depth only slightly increased the capture of CpG sites and the inflection point is on 10× coverage, consistent with previous literature [15, 17]. Thus, the number of CpG sites with coverage ≥ 10× from MC-seq was used in subsequent analyses.

After quality control filtering, MC-seq captured an average of 2,878,207 methylation CpG sites with coverage ≥ 10× among the 12 DNA samples, with an average of 3,708,550 CpG sites among samples with high DNA input (> 1000 ng), an average of 3,046,172 CpG sites among samples with medium DNA input (300–1000 ng), and an average of 1,879,898 CpG sites among samples with low DNA input (150–300 ng) (Fig. 1c and Table 1). Despite the fact that the detected number of CpG sites varied depending on DNA input quantity, CpG-based correlation among the common CpG sites between samples with high and medium, high and low DNA input quantities exceeded *r* > 0.95. Correlations of common CpG sites between medium and low DNA inputs were also high with *r* in 0.92–0.94 (Table 2). Figure 1d shows the scatterplot of 10,000 randomly selected common CpGs between samples with high and medium, high and low, and medium and low DNA input quantities. Pair-wise participant-based correlations were high as *r* > 0.98 among common CpG sites (Table 3). Overall, MC-seq exhibited good reproducibility. The methylation profile generating in high DNA input from each participant was used for subsequent analyses.

**Table 1:** Detected CpG number by DNA amount in MC-seq with coverage ≥ 10×

DNA amount	Participant ID	CpG number	Average CpG number
Low	S1	1,774,940	1,879,898
	S2	1,831,086	
	S3	2,154,732	
	S4	1,758,834	
Medium	S1	2,768,456	3,046,172
	S2	3,338,200	
	S3	3,119,259	
	S4	2,958,772	
High	S1	3,406,879	3,708,550
	S2	3,642,776	
	S3	3,722,552	
	S4	4,061,994	
Total average			2,878,207

**Table 2:** Comparison of MC-seq between samples with high, medium, and low DNA input amount

Participant ID	DNA amount			
	High	Medium	Common CpG	Pearson correlation
S1	3,406,879	2,768,456	2,747,844	0.984
S2	3,642,776	3,338,200	3,283,296	0.984
S3	3,722,552	3,119,259	3,101,938	0.977
S4	4,061,994	2,958,772	2,957,239	0.979

	DNA amount			
	High	Low	Common CpG	Pearson correlation
S1	3,406,879	1,774,940	1,771,936	0.960
S2	3,642,776	1,831,086	1,829,919	0.966
S3	3,722,552	2,154,732	2,153,175	0.974
S4	4,061,994	1,758,834	1,758,622	0.963

	DNA amount			
	Medium	Low	Common CpG	Pearson correlation
S1	2,768,456	1,774,940	1,745,241	0.942
S2	3,338,200	1,831,086	1,827,536	0.943
S3	3,119,259	2,154,732	2,135,980	0.939
S4	2,958,772	1,758,834	1,744,416	0.928

**Table 3:** Overlap of detected CpG across samples with high DNA input amount by MC-seq

Participant ID 1	Participant ID 2	Common CpG	Pearson *R*
S1	S2	3,336,037	0.980
S1	S3	3,350,314	0.976
S1	S4	3,394,970	0.982
S2	S3	3,519,772	0.978
S2	S4	3,613,753	0.982
S3	S4	3,676,406	0.978

#### Distribution of methylome regions by MC-seq and EPIC

**Fig. 2:**
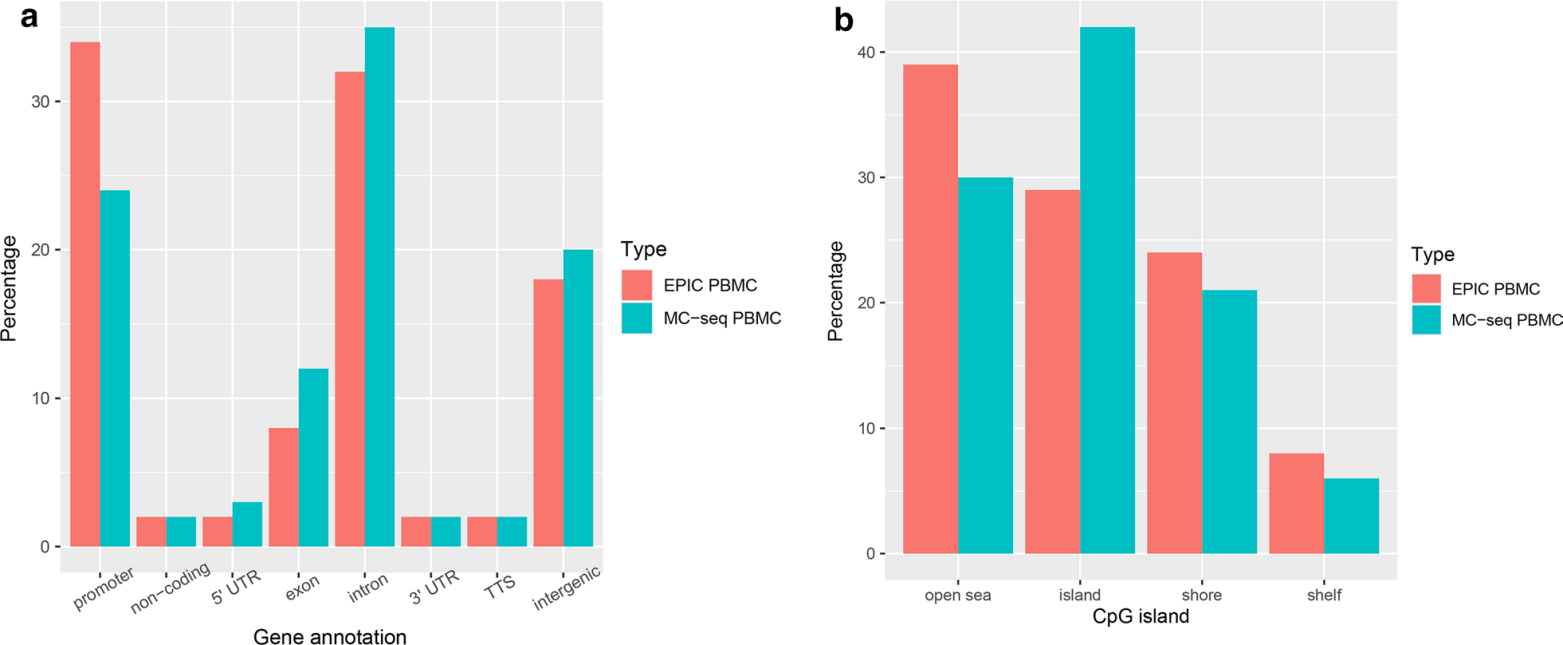
Comparison of CpG proportion in epigenomic regions between MC-seq and EPIC. **a** Distribution of genomic regions (intergenic, promoter, 5′UTR, exon, intron, non-coding, 3′UTR, transcription termination site (TTS), and non-coding). **b** Distribution of CpG position relative to CpG islands (CpG island, shore, shelf, and open sea)

We compared genome-wide DNA methylation captured by MC-seq and by EPIC array in the four high DNA input samples. An average of 3,708,550 CpG sites were detected by MC-seq and 846,464 CpG sites by EPIC array. Overall, MC-seq detected 11.5 times more CpG sites in exons and 10.2 times more CpG sites in 5′ UTR region compared to the EPIC array, and 4.8 to 8.9 times more CpG site in other categories of genomic regions by MC-seq compared to EPIC array. However, the proportion of CpGs out of all CpGs successfully measured that map to gene regions in MC-seq as compared to the EPIC array did not significantly differ between these two platforms. For example, the proportion of CpG sites in transcription termination site (TTS) regions was similar between two platforms. MC-seq showed slightly greater proportions of CpG sites in 5′UTR and exon regions, while the EPIC array detected a greater proportion of CpG sites in promoter regions (Fig. 2a). In terms of CpG sites in relation to CpG islands including open seas, shelves, and shores, MC-seq detected 10.9 times more CpG sites located on CpG islands and 5.4–6.2 times more on other regions compared with the EPIC array. The proportion of CpG islands detected by MC-seq was greater than by the EPIC array (42% versus 29%), while the EPIC array detected a modestly higher percentage of CpG sites located in open seas than the MC-seq (39% versus 31%) (Fig. 2b).

#### Comparison of Common CpG sites Measured by MC-seq and EPIC

**Fig. 3:**
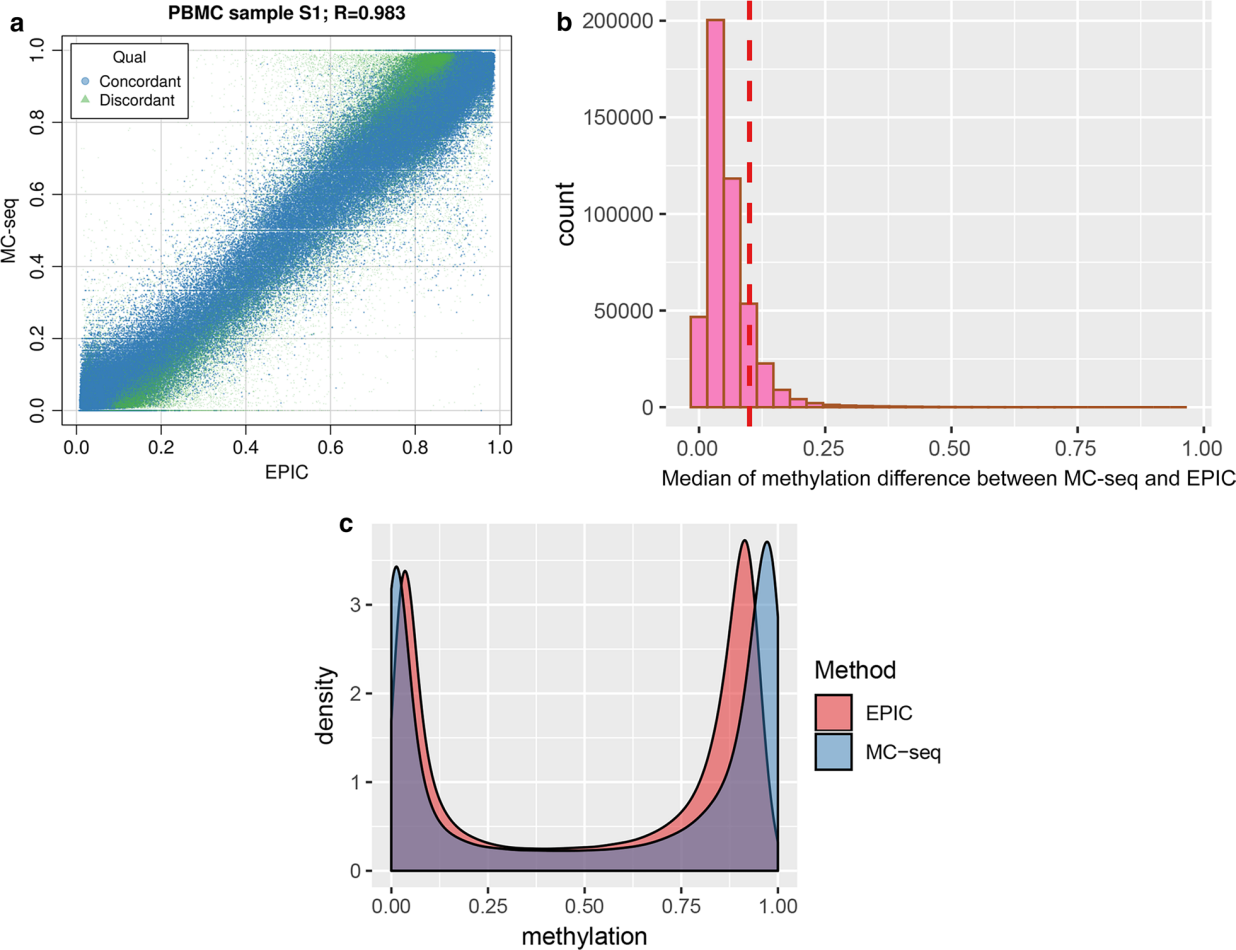
Comparing methylation values among common CpG sites between MC-seq and EPIC. **a** Correlation of methylation values measured by MC-seq and EPIC array among common CpG sites in participant S1. Blue dots represent concordant CpGs with Δ*β* < 0.1 between the two platforms and green dots represent discordant quality with Δ*β* ≥ 0.1; **b** The distribution of median Δ*β* in common CpG sites between MC-seq and EPIC array. The red dotted line represents Δ*β* = 0.1 as a cutoff for concordant CpG site between two platforms. **c** The density plot of methylation values among common CpG sites profiled by MC-seq and EPIC array in participant S1

A total of 472,540 CpG sites were measured by both platforms. Overall, the correlations of these shared CpG sites was high, ranging from *r* = 0.983 to 0.985 across the four samples (Fig. 3a). Figure 3b presents the distribution of the absolute difference of methylation *β* values between MC-seq and EPIC. A small proportion of CpG sites (1.4%) were discordant (i.e., Δ*β* > 0.1), while 98.6% of CpG sites were concordant (i.e., Δ*β* < 0.1). Figure 3a presents the concordant (blue) and discordant CpG sites (green) between MC-seq and EPIC for participant S1 (Fig. 3a). The 60,753 discordant CpG sites appeared to be randomly distributed across the epigenome (Additional file 1: Figure S1). Among the discordant CpG sites, we identified 239 CpG sites with highly discrepant methylation (i.e., Δ*β* > 0.5) (Table 4). Addition file 2: Table S1 presents top 100 discordant CpG sites with medium discrepant methylation (D*β* = 0.1 ~ 0.4)Additional file 3: Figure S2 shows that participants S2, S3, and S4 have similar distribution of concordant and discordant plots as participant S1.

Density plots of methylation *β* showed bimodal distribution using both the MC-seq and the EPIC array platforms (Fig. 3c). Density of methylated CpG sites was slightly higher than the density of unmethylated CpG sites on both platforms. However, the two peaks in the EPIC array density plot were closer than the two peaks in the MC-seq density plot (Fig. 3c), indicating that MC-seq captures a higher dynamic range (i.e., more methylated and unmethylated) of CpG sites than the EPIC array. Additional file 4: Figure S3 shows that participants S2, S3, and S4 have similar density plots.

## Discussion

We profiled the same PBMC samples using the MC-seq and EPIC array platforms and compared their performance. Our results show that the Agilent SureSelect Methyl-Seq targeted enrichment platform produced high-quality DNA methylation sequencing data at single base-pair resolution. MC-seq can reliably detect CpG sites with DNA input quantities as low as 300 ng. Overall, MC-seq detected 3–4 times more CpG sites than the EPIC array; however, the proportion of CpG sites mapped on functional genomic regions was similar between the two platforms. Methylation at a majority of CpG sites between the two platforms was highly correlated, while methylation at a low percentage of CpG sites differed significantly between the two platforms. Specifically, we found that methylation at 239 CpG sites differed significantly between the two platforms with absolute Δ*β* values greater than 0.5, which suggests that these CpG sites should be interpreted with caution in EWAS studies.

Our results show that MC-seq produces highly reliable CpG site methylation estimates across the genome. The observed CpG-based reproducibility is high, suggesting that technical variation on CpG calls is low. Inter-personal methylation variation is important for EWAS analysis. We found that our participant-based methylation on common CpG sites across four participants is also highly correlated, which further demonstrates the high reproducibility of this platform.

One disadvantage of sequencing-based approaches is the requirement for a larger quantity of input DNA than array-based approaches for methylation profiling. The recommended input DNA for Agilent SureSelect platform is 1ug, while input DNA quantity for EPIC array can be as low as 250 ng. Input DNA quantity is one important consideration influencing study design and methylation assay platform selection for population-based EWAS. Agilent has reported that DNA quantity can be as low as 250 ng for SureSelect sequencing [14]. To examine whether DNA quantity impacts the performance of MC-seq and to test whether low input DNA quantity also produces reliable CpG detection, we compared the capacity of CpG site detection across three different DNA input quantities. We found that medium DNA input quantity (i.e., 300 ng to 1000 ng) reliably detected CpG sites is comparable to the number of CpG sites captured by high DNA input quantity (i.e., greater than 1000 ng). Low DNA input quantity (i.e., less than 300 ng) detected the lowest number of CpG sites compared with high and medium DNA input quantity. For samples with low DNA input quantity, additional PCR cycles are needed to ensure post-capture library yield that results in extensive duplicate reads. In the four low DNA input samples, the duplicate rate exceeds 80%. Thus, removing duplicate reads is an important step in the QC process for MC-seq. We found that the number of CpG sites in low DNA input samples without duplicated reads still is significantly higher than the number of CpG sites detected by the EPIC array.

Consistent with previous reports, we found that methylation at the majority of CpG sites measured by both approaches (> 98%) is highly consistent between MC-seq and array-based methods. However, we identified 1.4% of CpG sites with discrepancies in CpG methylation that exceeds 10%. More importantly, 239 out of 60,753 discordant CpG sites had methylation differences exceeding 50%. These CpG sites are located on 159 gene regions (Table 4). Some of these genes have been previously reported to be associated with diseases. For example, *SLC45A4* was reported to harbor an epigenetic marker for adiposity [21]. The methylation *β* differs on the CpG site of this gene by as much as 0.63 between the two platforms. We have also identified those CpG sites that showed less but still apparent discrepancy between the two assay platforms (i.e., absolute difference of beta values between 0.1 and 0.5). The top 100 CpG sites discrepant in a range of 0.1–0.4 between two platforms are presented in Table S2 to allow investigators to consider this potential source of bias in EWAS findings. The discrepancy might be due to bias in the performance of the beadchip assay at these positions, sequence context-dependent impacts on the performance of sequencing, batch effects, or a combination of these possibilities. This large discrepancy warrants further investigation and interpretation of findings at these CpG sites must be interpreted with caution.

**Table 4:** Discordant CpG sites between MC-seq and EPIC (difference>0.5)

Probe	Chr	Position	Gene	MC-seq median	EPIC median	Median Difference between MC-seq and EPIC	Refgene group	Relation to CpG island
cg09156519	9	103361572		0.009	0.960	0.95		S_Shore
cg18176117	9	96097296	*C9orf129*	0.000	0.932	0.93	Body	N_Shore
cg14268958	10	133453066		0.000	0.899	0.89		S_Shelf
cg10576280	10	124133822	*PLEKHA1*	0.072	0.948	0.88	TSS1500	N_Shore
cg01005486	3	13246006		0.047	0.886	0.84		Island
cg23433318	19	667542		0.007	0.866	0.84		Island
cg10766172	7	27498479		0.989	0.147	0.82		
cg11812439	4	68928706	*LOC550113*;*SYT14P1*;*TMPRSS11F*	0.973	0.155	0.82	Body; Body; Body	
cg23950473	5	154393265	*KIF4B*; *KIF4B*	0.992	0.174	0.82	1stExon; 5’UTR	
cg00259849	8	4183880	*CSMD1*	0.000	0.821	0.82	Body	
cg23981150	1	161111090		1.000	0.217	0.78		Island
cg09698465	12	133000178		0.906	0.080	0.78		Island
cg20450977	11	10529463	*MTRNR2L8*; *MTRNR2L8*	0.964	0.189	0.77	3’UTR; 1stExon	
cg01053463	1	26186087	*C1orf135*	0.021	0.757	0.76	TSS1500	Island
cg12499827	2	202004893	*CFLAR*; *CFLAR*; *CFLAR*; *CFLAR*; *CFLAR*; *CFLAR*; *CFLAR*; *CFLAR*; *CFLAR*	0.971	0.215	0.76	TSS200; Body; Body; Body; Body; Body; Body; Body; Body	
cg03133777	2	170361364	*BBS5*	1.000	0.244	0.75	3’UTR	
cg19040702	17	22023833		0.969	0.230	0.75		
cg21675871	11	69813397		0.217	0.960	0.74		Island
cg04240493	3	148414664	*AGTR1*; *AGTR1*; *AGTR1*; *AGTR1*	0.979	0.258	0.72	TSS1500; TSS1500; TSS1500; TSS1500	N_Shore
cg16889427	10	127584375	*FANK1*	0.036	0.759	0.71	TSS1500	Island
cg25916505	18	32820654	*ZNF397*; *ZNF397*	0.000	0.711	0.71	TSS1500; TSS1500	N_Shore
cg13525026	17	18061071	*MYO15A*	1.000	0.290	0.71	Body	
cg07825433	4	1215099	*CTBP1*; *CTBP1*	0.000	0.716	0.71	Body; Body	N_Shelf
cg03846641	2	109746751	*LOC100287216*; *SH3RF3*	0.239	0.952	0.71	TSS200; Body	Island
cg19188207	2	10340837	*C2orf48*	1.000	0.290	0.71	Body	
cg11495544	17	73402155	*GRB2*; *GRB2*	0.753	0.049	0.70	TSS1500; TSS1500	S_Shore
cg06931905	8	42036940	*PLAT*; *PLAT*	0.896	0.197	0.70	Body; Body	
cg03348902	1	569603		0.869	0.168	0.70		
cg27120934	6	129480619	*LAMA2*; *LAMA2*	0.979	0.297	0.69	Body; Body	
cg07576219	1	55012408	*ACOT11*; *ACOT11*	0.927	0.250	0.69	TSS1500; TSS1500	S_Shelf
cg08400246	5	156570642	*MED7*; *MED7*	0.153	0.870	0.68	TSS1500; TSS1500	S_Shore
cg27626141	8	103876469	*AZIN1*; *AZIN1*	0.000	0.682	0.68	TSS200; TSS200	Island
cg26688472	2	203638928	*ICA1L*	0.984	0.303	0.68	3’UTR	Island
cg26101183	10	65930786		0.957	0.279	0.68		Island
cg27090007	13	28519388	*ATP5EP2*	0.985	0.321	0.67	Body	
cg11896012	19	53696753	*ZNF665*	0.048	0.700	0.67	TSS200	S_Shore
cg02606018	12	10658281		0.979	0.323	0.66		
cg00438164	4	100870480	*H2AFZ*; *LOC256880*	0.004	0.650	0.65	Body; TSS1500	Island
cg21164300	9	136098495		0.000	0.644	0.64		N_Shelf
cg15891076	10	65930618		0.971	0.328	0.64		Island
cg05948389	5	1641924		0.014	0.660	0.64		N_Shelf
cg10507965	10	102107251	*SCD*; *SCD*	0.011	0.642	0.64	5’UTR; 1stExon	Island
cg21662326	11	14521493	*COPB1*; *COPB1*; *COPB1*	0.643	0.012	0.63	TSS200; TSS200; TSS200	
cg09646578	8	5019363		0.310	0.934	0.63		
cg24717964	20	61477008	*DPH3B*; *DPH3B*; *TCFL5*	0.986	0.356	0.63	1stExon; 5’UTR; Body	
cg07437919	8	142234483	*SLC45A4*	0.957	0.313	0.63	Body	N_Shore
cg01105403	2	240723304		0.050	0.890	0.63		
cg20482143	7	64340804		0.982	0.346	0.63		
cg11187452	22	49698612		0.017	0.653	0.63		Island
cg24504954	3	61237217	*FHIT*; *FHIT*	0.017	0.649	0.62	TSS200; TSS200	Island
cg27434351	11	14521491	*COPB1*; *COPB1*; *COPB1*	0.639	0.016	0.62	TSS200; TSS200; TSS200	
cg15864074	2	120974042		0.976	0.354	0.62		
cg00913521	12	89893799	*WDR51B*	0.977	0.339	0.62	Body	
cg27534567	1	568536		0.834	0.262	0.62		
cg24515136	17	49024834		0.949	0.328	0.62		S_Shelf
cg01417615	1	52456419	*RAB3B*	0.629	0.015	0.61	TSS200	Island
cg00236302	12	69004867	*RAP1B*; *RAP1B*	0.000	0.612	0.61	5’UTR; 5’UTR	Island
cg10747603	22	29197018	*XBP1*; *XBP1*	0.022	0.627	0.61	TSS1500; TSS1500	S_Shore
cg03594447	1	20359744		1.000	0.358	0.61		
cg23045277	4	190587808		0.299	0.910	0.61		
cg02218809	16	29973300	*TMEM219*; *TMEM219*	0.020	0.612	0.61	TSS200; TSS200	Island
cg05646491	10	135379754	*SYCE1*; *SYCE1*; *SYCE1*	0.988	0.382	0.60	TSS1500; 5’UTR; TSS1500	Island
cg07596174	20	55926107	*RAE1*; *RAE1*	0.014	0.613	0.60	TSS1500; TSS200	N_Shore
cg03543448	16	4384967	*GLIS2*	0.927	0.315	0.60	Body	
cg25793197	5	31923469	*PDZD2*	0.976	0.379	0.60	Body	
cg21392229	2	161223778	*RBMS1*; *RBMS1*	1.000	0.384	0.60	Body; Body	
cg05607320	12	53342553	*KRT18*; *KRT18*	0.064	0.651	0.60	TSS200; TSS1500	N_Shore
cg13896861	9	94878241	*SPTLC1*; *SPTLC1*	0.117	0.711	0.60	TSS1500; TSS1500	S_Shore
cg03064900	4	190566141		0.323	0.921	0.60		N_Shore
cg16199859	3	75263685		0.861	0.276	0.60		
cg15006843	1	205720633	*NUCKS1*	0.880	0.260	0.60	TSS1500	S_Shore
cg02498218	4	26361371	*RBPJ*; *RBPJ*; *RBPJ*; *RBPJ*	0.979	0.388	0.59	Body; Body; 5’UTR; Body	Island
cg07116712	15	96887959		0.091	0.681	0.59		Island
cg11643306	20	34204831	*SPAG4*	0.038	0.630	0.59	Body	S_Shore
cg08568561	7	42834498		0.981	0.392	0.59		
cg06669598	6	127622363	*ECHDC1*; *ECHDC1*; *ECHDC1*; *ECHDC1*; *ECHDC1*	0.984	0.351	0.59	3’UTR; 3’UTR; Body; Body; Body	
cg22805431	3	113955600	*ZNF80*	0.983	0.409	0.59	1stExon	
cg24636332	17	4437925	*SPNS2*	0.301	0.939	0.59	Body	N_Shore
cg05924191	15	35279830	*ZNF770*	0.008	0.605	0.59	5’UTR	N_Shore
cg14402194	14	23398944	*PRMT5*; *PRMT5*; *PRMT5*; *PRMT5*; *PRMT5*; *PRMT5*; *LOC101926933*	0.028	0.592	0.58	TSS200; TSS200; TSS200; TSS200; TSS200; TSS200; Body	S_Shore
cg01737532	4	190862170	*FRG1*	0.000	0.584	0.58	1stExon	Island
cg25744017	15	52819324	*MYO5A*; *MYO5A*	0.957	0.379	0.58	Body;Body	N_Shore
cg03432151	15	89745000	*ABHD2*; *ABHD2*	0.948	0.360	0.58	3’UTR; 3’UTR	
cg27196695	10	134571377	*INPP5A*	1.000	0.420	0.58	Body	
cg27571351	10	17619364		0.986	0.407	0.58		
cg02775804	2	120974080		0.977	0.401	0.58		
cg16461530	10	134798264		0.664	0.106	0.58		
cg12654770	10	52487693		0.962	0.385	0.58		
cg16112880	1	201123745	*TMEM9*	0.003	0.579	0.58	TSS200	Island
cg20641423	8	125315065		0.911	0.338	0.57		S_Shore
cg23248615	10	2005709		0.905	0.313	0.57		
cg25550279	7	53254983		0.965	0.381	0.57		Island
cg01070250	1	569687		0.843	0.271	0.57		
cg06977575	4	139481990		0.953	0.376	0.57		Island
cg14511644	9	15055021		0.977	0.399	0.57		
cg08947542	8	35383200	*UNC5D*	0.879	0.309	0.57	Body	
cg10258063	2	217363243	*RPL37A*	0.043	0.613	0.57	TSS1500	N_Shore
cg24209723	18	12913133		0.973	0.399	0.57		S_Shore
cg02265379	5	87898506	*LOC645323*	0.971	0.384	0.57	Body	Island
cg18925601	7	158752715		0.006	0.574	0.57		Island
cg01406075	11	58731104		0.885	0.309	0.57		N_Shore
cg13545297	12	54404315	*HOXC8*	0.229	0.791	0.57	Body	S_Shore
cg09036531	10	96991505		0.968	0.402	0.57		
cg25649283	9	140714075	*EHMT1*	0.382	0.950	0.57	Body	Island
cg06204030	17	7792051	*CHD3*; *CHD3*; *CHD3*	0.761	0.141	0.57	TSS200; TSS200; Body	S_Shelf
cg18627328	19	621561	*POLRMT*	0.980	0.411	0.56	Body	Island
cg13085681	8	48920761	*UBE2V2*	0.009	0.576	0.56	TSS1500	N_Shore
cg00999469	6	25107287	*CMAHP*	0.043	0.931	0.56	Body	
cg20960039	9	130213605	*LRSAM1*; *RPL12*; *LRSAM1*; *LRSAM1*; *LRSAM1*; *RPL12*	0.020	0.582	0.56	TSS1500; 1stExon; TSS200; TSS200; TSS200; 5’UTR	Island
cg04400841	2	208988863	*CRYGD*	0.215	0.764	0.56	Body	Island
cg12476298	19	58426697	*ZNF417*	0.977	0.407	0.56	Body	N_Shore
cg23997402	19	14275669	*LPHN1*; *LPHN1*	0.972	0.442	0.56	Body; Body	S_Shore
cg16935370	5	154393281	*KIF4B*; *KIF4B*	0.981	0.414	0.56	1stExon; 5′UTR	
cg04222159	1	204981786	*NFASC*; *NFASC*; *NFASC*; *NFASC*	0.630	0.073	0.56	Body; Body; Body; Body	
cg06396237	8	120779442	*TAF2*	0.985	0.430	0.56	Body	
cg06599543	6	165749446	*PDE10A;PDE10A*	0.857	0.332	0.56	Body;Body	S_Shore
cg11566832	10	88659593	*BMPR1A*	0.145	0.693	0.55	Body	
cg20334010	15	41047916	*RMDN3*; *RMDN3*	0.020	0.570	0.55	TSS1500; TSS1500	S_Shore
cg18245781	5	3659697		0.283	0.852	0.55		
cg03761810	2	10264850	*RRM2*; *RRM2*	0.018	0.567	0.55	Body; Body	S_Shore
cg02122372	3	149657597	*RNF13*; *RNF13*	1.000	0.439	0.55	Body; Body	
cg06753227	18	9475508	*RALBP1*	0.000	0.553	0.55	TSS200	Island
cg14131834	13	45914250	*LOC100190939*; *TPT1*	0.040	0.594	0.55	TSS1500; Body	N_Shore
cg25583180	5	177614382	*GMCL1L*	1.000	0.451	0.55	Body	Island
cg09112623	6	33756905	*LEMD2*; *LEMD2*	0.568	0.019	0.55	5’UTR; 1stExon	Island
cg11759477	4	190861959	*FRG1*	0.000	0.548	0.55	TSS200	Island
cg12796755	14	51132292	*SAV1*	0.932	0.383	0.55	Body	N_Shelf
cg19693446	14	102144192		0.958	0.407	0.55		
cg25187648	3	49395165	*GPX1*; *GPX1*; *GPX1*	0.018	0.568	0.55	Body; 3’UTR; 1stExon	Island
cg20391833	6	167116208	*RPS6KA2*	0.964	0.431	0.55	Body	
cg09705232	6	97611802	*MIR548H3*; *C6orf167*	0.974	0.428	0.55	Body; Body	
cg04643437	12	14518655	*ATF7IP*; *ATF7IP*	0.000	0.561	0.55	1stExon; 5’UTR	Island
cg17558062	13	45965415	*LOC100190939*	1.000	0.456	0.54	Body	Island
cg26825848	4	190566175		0.350	0.899	0.54		N_Shore
cg13943141	9	93205862		0.846	0.296	0.54		
cg26951705	19	56612697	*ZNF787*	0.000	0.542	0.54	Body	Island
cg24654094	1	160340832	*NHLH1*	0.964	0.433	0.54	Body	Island
cg02996355	14	81879375		0.909	0.364	0.54		
cg11914812	12	56904792		1.000	0.459	0.54		
cg24895977	19	35861796		0.990	0.450	0.54		
cg11637682	6	147124984	*LOC729176*; *C6orf103*	0.867	0.300	0.54	TSS200; Body	
cg07089633	14	73396378	*DCAF4*; *DCAF4*; *DCAF4*; *DCAF4*; *DCAF4*	1.000	0.450	0.54	5’UTR; 5’UTR; 5’UTR; 5’UTR; 5’UTR	S_Shelf
cg20360416	4	7246127	*SORCS2*	0.018	0.659	0.54	Body	
cg25627920	17	39992620	*NT5C3B*; *NT5C3B*; *NT5C3B*; *KLHL10*	0.016	0.552	0.54	TSS200; TSS200; TSS200; TSS1500	Island
cg24000259	5	55488291	*ANKRD55*	0.961	0.411	0.54	Body	
cg09138437	11	64527189	*PYGM*; *PYGM*	0.993	0.445	0.54	1stExon; 1stExon	
cg02673636	1	109647056		0.976	0.437	0.54		S_Shelf
cg18740872	5	39220260	*FYB*; *FYB*	1.000	0.466	0.53	TSS1500; TSS1500	
cg14354292	7	63353606		0.960	0.426	0.53		
cg17704839	19	9939038	*UBL5; UBL5*	0.014	0.542	0.53	Body; Body	S_Shore
cg05971373	7	157498604	*PTPRN2*; *PTPRN2*; *PTPRN2*	1.000	0.467	0.53	Body; Body; Body	S_Shelf
cg05291429	17	1494566	*SLC43A2*	0.402	0.969	0.53	Body	S_Shelf
cg08841342	3	156528470	*PA2G4P4*	0.976	0.444	0.53	Body	
cg04096697	6	37012867		0.983	0.451	0.53		Island
cg26878995	1	168106731	*GPR161*	0.052	0.570	0.53	TSS1500	S_Shore
cg24031524	20	19804606		0.990	0.468	0.53		
cg19311470	4	39460490	*RPL9*; *RPL9*; *LIAS*; *LIAS*	0.004	0.529	0.53	TSS1500; 5’UTR; TSS200; TSS200	Island
cg02181482	5	178942685		0.956	0.449	0.53		
cg05346902	19	47910374	*MEIS3*; *MEIS3*	0.068	0.593	0.53	Body; Body	Island
cg16470772	10	8203304		0.971	0.445	0.53		
cg10115022	1	27527942		0.974	0.464	0.53		Island
cg27231717	6	26319377		0.905	0.386	0.53		
cg09451549	19	8386408	*RPS28*; *NDUFA7*; *RPS28*	0.000	0.527	0.53	5’UTR; TSS200; 1stExon	Island
cg07628841	2	27851430	*GPN1*; *CCDC121*; *GPN1*; *GPN1*; *CCDC121*; *CCDC121*; *GPN1*; *GPN1*	0.010	0.536	0.53	TSS200; 1stExon; TSS200; TSS1500; 5’UTR; 1stExon; TSS200; TSS1500	
cg03816081	10	29577743	*LYZL1*	0.863	0.301	0.52	TSS1500	
cg00762003	21	45393541	*AGPAT3*; *AGPAT3*	0.383	0.892	0.52	Body; Body	Island
cg19466922	7	130138026	*MEST*; *MEST*; *MEST*; *MEST*; *MEST*; *MEST*	1.000	0.476	0.52	Body; Body; Body; Body; Body; Body	
cg07712165	17	80899280	*TBCD*	0.440	0.959	0.52	Body	Island
cg01199952	13	25591486		0.984	0.456	0.52		N_Shore
cg11374834	3	75263691		0.950	0.428	0.52		
cg02974491	1	1162280	*SDF4*; *SDF4*	0.403	0.964	0.52	Body; Body	Island
cg10555853	1	33516627		0.929	0.407	0.52		Island
cg21216606	2	207275704		0.985	0.464	0.52		
cg17711541	6	26124704	*HIST1H2AC*; *HIST1H2BC*	0.007	0.529	0.52	1stExon; TSS1500	Island
cg06412823	7	22541074	*STEAP1B*; *STEAP1B*	0.196	0.739	0.52	TSS1500; TSS1500	S_Shore
cg03054343	11	50238214		1.000	0.463	0.52		Island
cg05766605	1	19384827		0.423	0.938	0.52		
cg07684215	10	132976057	*TCERG1L*	0.181	0.923	0.52	Body	
cg27193858	6	41169120	*TREML2*	0.181	0.691	0.52	TSS200	
cg00964321	16	15083956	*PDXDC1*	0.906	0.386	0.52	Body	Island
cg25394572	11	56457777	*OR8U8*	0.949	0.429	0.52	Body	
cg10667969	3	149181941		0.967	0.458	0.52		
cg18394854	5	8457818		0.212	0.732	0.52		Island
cg05741225	10	133917303	*JAKMIP3*	0.906	0.370	0.52	TSS1500	
cg06026769	12	20704492	*PDE3A*	0.993	0.473	0.52	Body	N_Shore
cg09032630	6	27831956	*HIST1H2AL*	0.802	0.311	0.52	TSS1500	N_Shore
cg16626480	22	25575426	*KIAA1671*	0.950	0.422	0.52	Body	Island
cg24534731	17	36888147	*CISD3*	0.969	0.457	0.52	Body	S_Shore
cg16202259	14	104625420	*KIF26A*	0.058	0.962	0.52	Body	Island
cg25325592	8	1439535		0.408	0.923	0.52		N_Shore
cg00391025	3	100427239	*TFG*; *TFG*	1.000	0.477	0.52	TSS1500; TSS1500	N_Shore
cg25149037	17	39736213		0.820	0.454	0.52		
cg19120749	11	1431650	*BRSK2*; *BRSK2*; *BRSK2*; *BRSK2*; *BRSK2*; *BRSK2*	0.468	0.983	0.52	TSS1500; TSS200; Body; Body; Body; Body	Island
cg24270624	10	95721318	*PIPSL*	0.947	0.421	0.52	Body	
cg16346588	10	242978	*ZMYND11*; *ZMYND11*; *ZMYND11*	0.970	0.459	0.51	Body; Body; Body	
cg02750322	15	83673816	*C15orf40*; *C15orf40*; *C15orf40*; *C15orf40*; *C15orf40*	0.966	0.451	0.51	Body; 3’UTR; Body; Body; Body	
cg04363536	3	49466872	*NICN1*	0.000	0.515	0.51	TSS200	S_Shore
cg17883371	1	91359225		1.000	0.480	0.51		Island
cg25018832	1	564471	*LOC101928626*	0.602	0.088	0.51	TSS200	
cg16838729	4	43901032		0.903	0.394	0.51		
cg23222247	17	47302219	*PHOSPHO1*; *PHOSPHO1*	0.009	0.531	0.51	Body;Body	Island
cg19496566	19	48249018	*GLTSCR2*	0.009	0.535	0.51	1stExon	Island
cg03165426	7	30726958	*CRHR2*; *CRHR2*	0.429	0.942	0.51	Body; 5’UTR	
cg19600494	2	106959525		0.968	0.455	0.51		Island
cg10854807	17	79479308	*ACTG1*	0.004	0.522	0.51	Body	Island
cg20699097	11	111957680	*TIMM8B*; *TIMM8B*; *SDHD*	0.009	0.524	0.51	TSS200; TSS200; 1stExon	Island
cg22819767	10	11866910	*C10orf47*	0.958	0.440	0.51	5’UTR	S_Shore
cg20254251	8	144557206	*ZC3H3*	0.993	0.450	0.51	Body	
cg00590830	1	32385224	*PTP4A2*; *PTP4A2*; *PTP4A2*; *PTP4A2*; *PTP4A2*	0.971	0.437	0.51	1stExon; 1stExon; 5’UTR; 5’UTR; 5’UTR	
cg03877767	2	11680057	*GREB1*; *GREB1*	0.172	0.683	0.51	5’UTR; TSS200	
cg00487526	15	90818384		0.956	0.444	0.51		Island
cg17501384	2	217364031	*RPL37A*	0.017	0.521	0.51	Body	S_Shore
cg17646418	6	166911767	*RPS6KA2*; *RPS6KA2*	0.987	0.468	0.51	Body; Body	
cg06757405	5	140789450	*PCDHGA4*; *PCDHGA9*; *PCDHGA1*; *PCDHGB1*; *PCDHGB6*; *PCDHGB6*; *PCDHGB3*; *PCDHGA6*; *PCDHGA8*; *PCDHGA5*; *PCDHGB4*; *PCDHGA3*; *PCDHGA2*; *PCDHGB2*; *PCDHGA7*; *PCDHGB5*	0.108	0.607	0.51	Body; Body; Body; Body; 1stExon; 1stExon; Body; Body; Body; Body; Body; Body; Body; Body; Body; Body	Island
cg13448596	8	2031599	*MYOM2*	0.381	0.887	0.51	Body	
cg16711165	11	111957658	*TIMM8B*; *TIMM8B*; *SDHD*	0.017	0.522	0.51	TSS200; TSS200; 1stExon	Island
cg16786640	4	3485263	*DOK7*; *DOK7*	0.447	0.954	0.51	Body; Body	N_Shore
cg07638938	10	131348599	*MGMT*	0.986	0.492	0.51	Body	
cg05407710	8	143329409	*TSNARE1*	0.960	0.451	0.51	Body	N_Shelf
cg07869994	3	174095190		0.988	0.482	0.51		Island
cg07455406	14	21077527		0.017	0.523	0.51		N_Shore
cg04576847	17	12623611	*MYOCD*; *MYOCD*; *MYOCD*; *MYOCD*	0.958	0.447	0.51	Body; 5’UTR; Body; 1stExon	
cg15699853	18	57684747		0.976	0.467	0.51		
cg11231240	8	82434638		1.000	0.482	0.51		Island
cg06157924	4	942005	*TMEM175*	0.451	0.957	0.51	Body	S_Shore
cg23679141	4	165118930	*MARCH1*; *ANP32C*	0.946	0.445	0.51	5’UTR; TSS200	
cg03053358	17	1029917	*ABR*; *ABR*	0.446	0.964	0.51	Body; 5’UTR	S_Shore
cg13705894	9	138305338		0.978	0.495	0.51		S_Shore
cg18512780	17	76117734	*TMC6*; *TMC6*	0.033	0.529	0.50	Body; Body	
cg06307940	16	46660818		0.986	0.487	0.50		
cg15541008	5	95297508	*ELL2*; *ELL2*	0.000	0.516	0.50	1stExon; 5’UTR	S_Shore
cg19969624	13	95954210	*ABCC4*; *ABCC4*; *ABCC4*; *ABCC4*	0.011	0.507	0.50	TSS1500; TSS1500; TSS1500; TSS1500	Island
cg01758870	7	23719630	*C7orf46*; *C7orf46*; *C7orf46*	0.028	0.545	0.50	TSS200;TSS200;TSS200	Island
cg08323201	15	101835348	*SNRPA1*	0.000	0.503	0.50	1stExon	Island
cg21863998	11	19770288	*NAV2*; *NAV2*; *NAV2*	0.439	0.905	0.50	Body; Body; Body	
cg11471802	8	47529015		0.348	0.882	0.50		Island
cg22281935	2	162934111		0.982	0.481	0.50		S_Shelf
cg06032540	15	43941563	*CATSPER2*; *CATSPER2*; *CATSPER2*	1.000	0.500	0.50	TSS1500; TSS1500; TSS1500	Island
cg18761878	1	568475		0.893	0.401	0.50		

One of the limitations of this study is the small number of participants used to estimate inter-sample variability. A previous study used a benchmark approach to evaluate performance of different platforms [17] and concluded that the EPIC array performed better than the MC-seq platform. However, the study did not remove duplicate reads as part of their data processing, which may have compromised the QC for MC-seq data processing as discussed above. Future studies, including benchmarking using a larger sample size, could further improve the analysis of platform performance. Of note, MC-seq detected high percentages of CHG and CHH sites across four methylome, which is consistent with previous reports [15]. The significances of those methylation sites warrant further investigation.

New approaches to measurement of DNA methylation continue to emerge that may warrant similar investigation in an ongoing effort to provide users with empiric comparisons to inform decisions about platform selection. One recent approach is enzymatic methyl-sequencing (EM-seq) (e.g., NEBNext EM-seq by New England Biolabs, Ipswich, MA) [22]. The input genomic DNA requirement is low 10–200 ng and EM-seq has comparable performance to WGBS [22], but its performance in relation to array- or capture sequencing-based approaches has not been reported. Should EM-seq gain popularity, it would be important to directly compare the performance of MC-seq and EM-seq to provide empiric evidence to users to inform platform selection.

Nevertheless, we have demonstrated that MC-seq is an efficient, reliable, and affordable platform that allows medium input quantity of DNA input (i.e., > 300 ng), which is equivalent to DNA input required for EPIC array. MC-seq has the advantage of capturing significantly more CpG sites than the EPIC array. Although methylation measurements between the two platforms are highly consistent, we have identified a small number of CpG sites that must be interpreted with caution if they are associated with a trait of interest because they showed significant discrepancies between the two platforms.

## Conclusions

Our results show that MC-seq is an efficient and reliable platform for methylome profiling with a broader coverage of the methylome than the array-based platform. Although methylation measurements in majority of CpGs are highly correlated, a number of CpG sites show large discrepancy between the two platforms, which warrants further investigation and needs cautious interpretation.

## Supplementary information

**Additional file 1: Figure S1.** A Manhattan plot showing the distribution of Δ*β* between MC-seq and EPIC array in PBMC by chromosome positions. Blue line represents Δ*β* = 0.1 and red line represents Δ*β* = 0.5.

**Additional file 2: Table S1.** Top 100 discordant CpG sites between MC-seq and EPIC array (Δ*β* = 0.1 ~ 0.4).

**Additional file 3: Figure S2.** Comparison of methylation values measured by MC-seq and EPIC array among common CpG sites in participant S2, S3, and S4. Blue dots represent concordant CpGs with Δ*β* < 0.1 between the two platforms and green dots represent discordant quality with Δ*β* ≥ 0.1

**Additional file 4: Figure S3.** The density plot of methylation values among CpG sites assayed in common by MC-seq and EPIC array in participant S2, S3, and S4

## Data Availability

All methylation data from MC-seq and EPIC platforms are deposited in GEO (GSE152922).

## Abbreviations

EM-seq: Enzymatic methyl-seq
EPIC: Illumina Infinium MethylationEPIC Beadchip
EWAS: Epigenome-wide association study
MC-seq: Methylation capture sequencing
PBMC: Peripheral blood mononuclear cell
PCR: Polymerase chain reaction
QC: Quality control
RTA: Real-time analysis
TTS: Transcription termination site
WGBS: Whole-genome bisulfite sequencing
