# Supplementary figures and images for: Comparison of methylation capture sequencing and Infinium MethylationEPIC array in peripheral blood mononuclear cells
Source: Epigenetics Chromatin. 2020 Nov 23;13:51. doi: 10.1186/s13072-020-00372-6 (PMC7684759; doi:10.1186/s13072-020-00372-6)

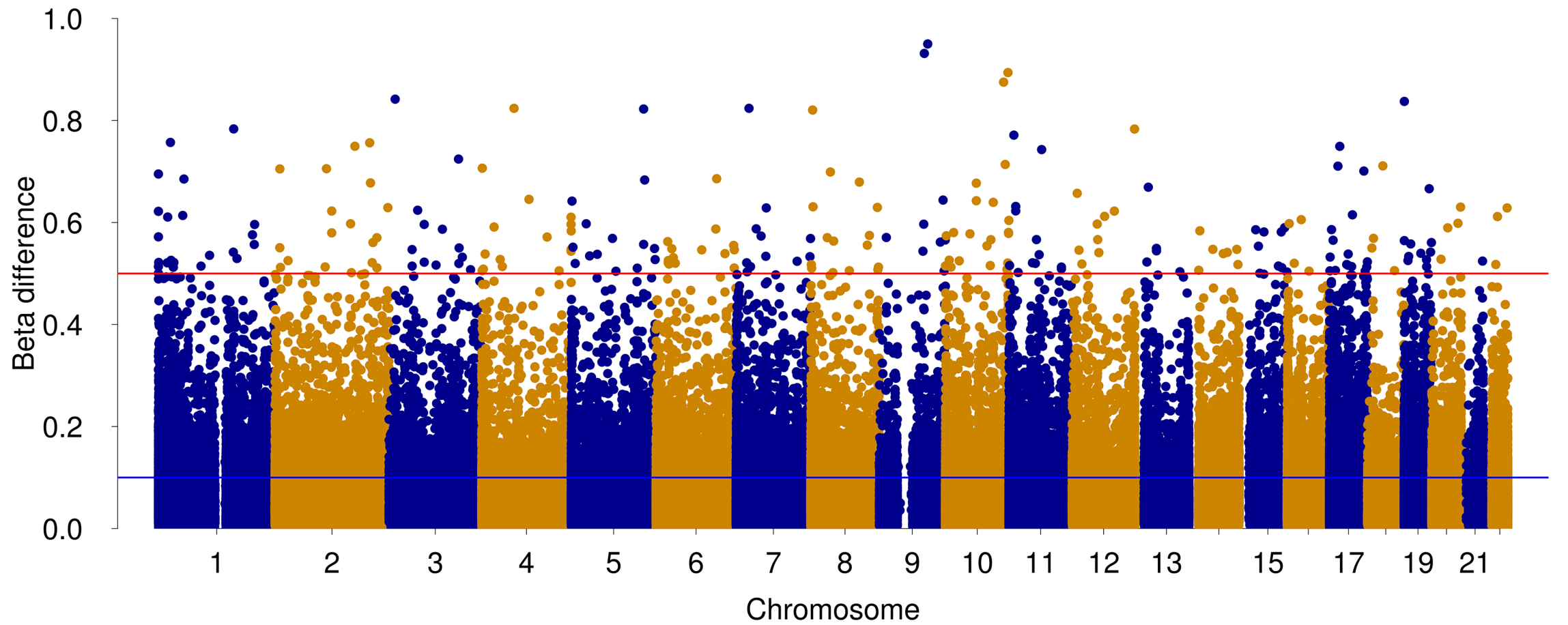

Supplement: Supplementary file 1 — Additional file 1: Figure S1. A Manhattan plot showing the distribution of Δβ between MC-seq and EPIC array in PBMC by chromosome positions. Blue line represents Δβ = 0.1 and red line represents Δβ = 0.5. [file 13072_2020_372_MOESM1_ESM.pdf]

**PBMC sample S2; R=0.985**

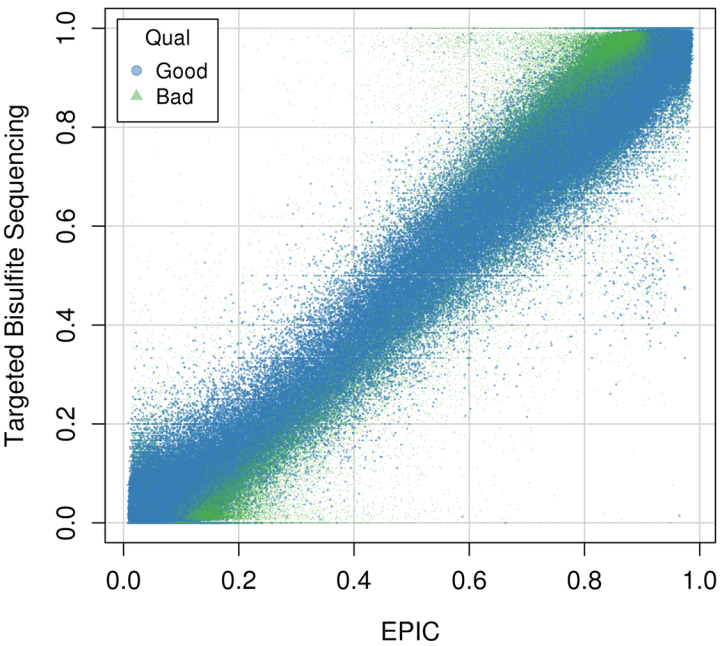

**PBMC sample S3; R=0.984**

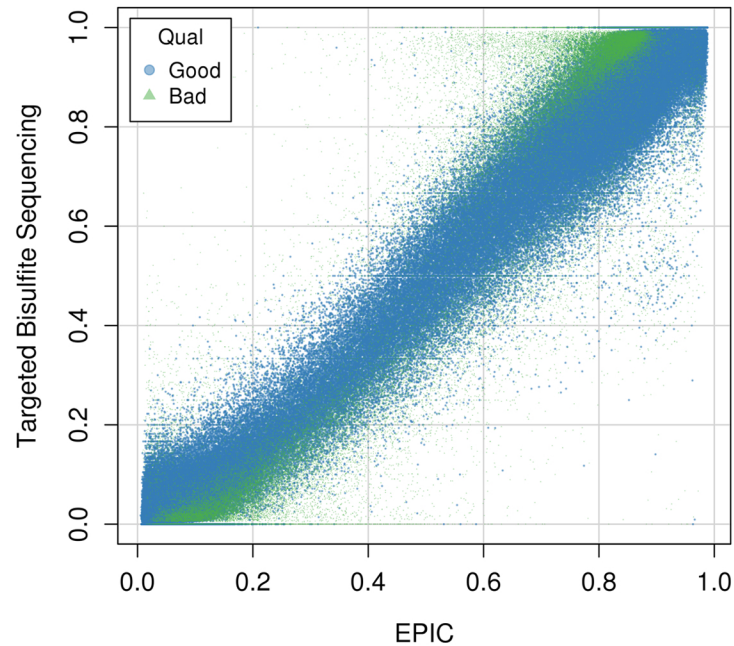

**PBMC sample S4; R=0.985**

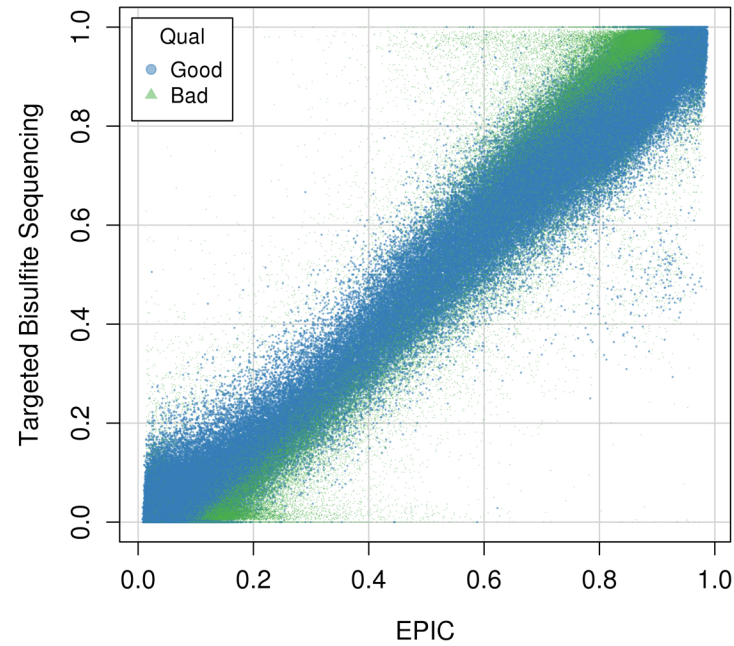

Supplement: Supplementary file 3 — Additional file 3: Figure S2. Comparison of methylation values measured by MC-seq and EPIC array among common CpG sites in participant S2, S3, and S4. Blue dots represent concordant CpGs with Δβ < 0.1 between the two platforms and green dots represent discordant quality with Δβ ≥ 0.1 [file 13072_2020_372_MOESM3_ESM.pdf]

PBMC sample S2

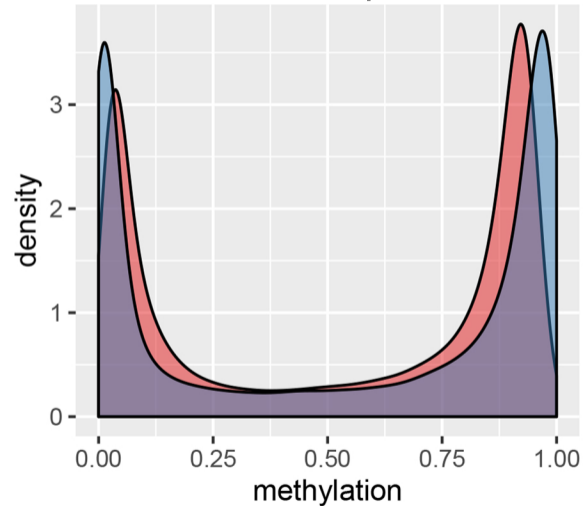

PBMC sample S3

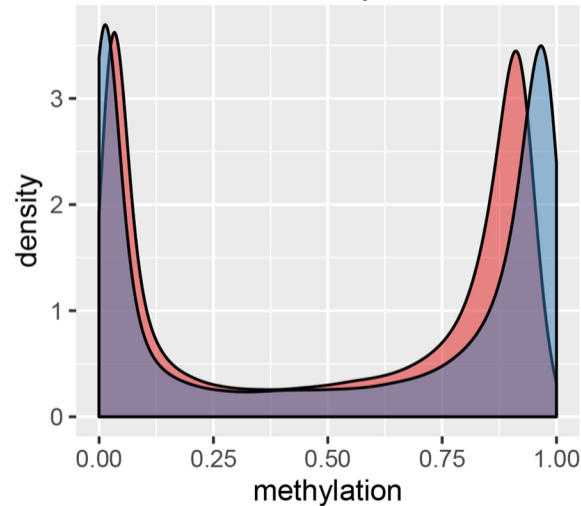

PBMC sample S4

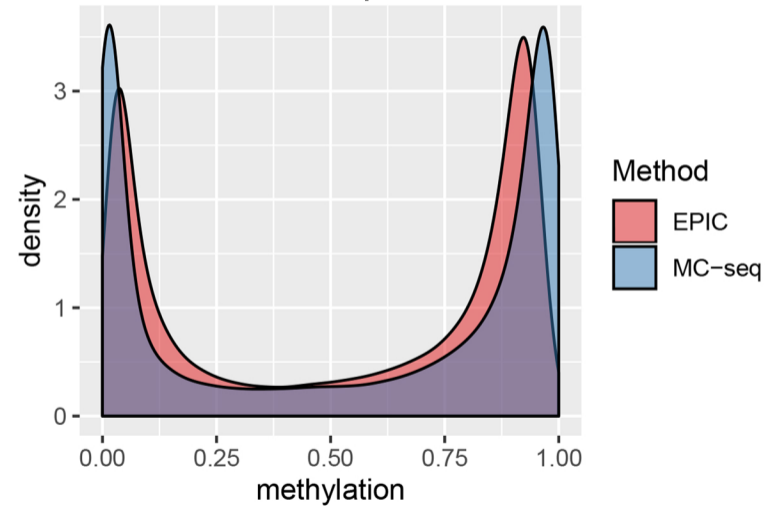

Supplement: Supplementary file 4 — Additional file 4: Figure S3. The density plot of methylation values among CpG sites assayed in common by MC-seq and EPIC array in participant S2, S3, and S4 [file 13072_2020_372_MOESM4_ESM.pdf]
